# Supplementary figures and images for: Toward Model-Informed Precision Dosing for Remimazolam: A Population Pharmacokinetic–Pharmacodynamic Analysis
Source: Pharmaceutics. 2024 Aug 26;16(9):1122. doi: 10.3390/pharmaceutics16091122 (PMC11435137; doi:10.3390/pharmaceutics16091122)

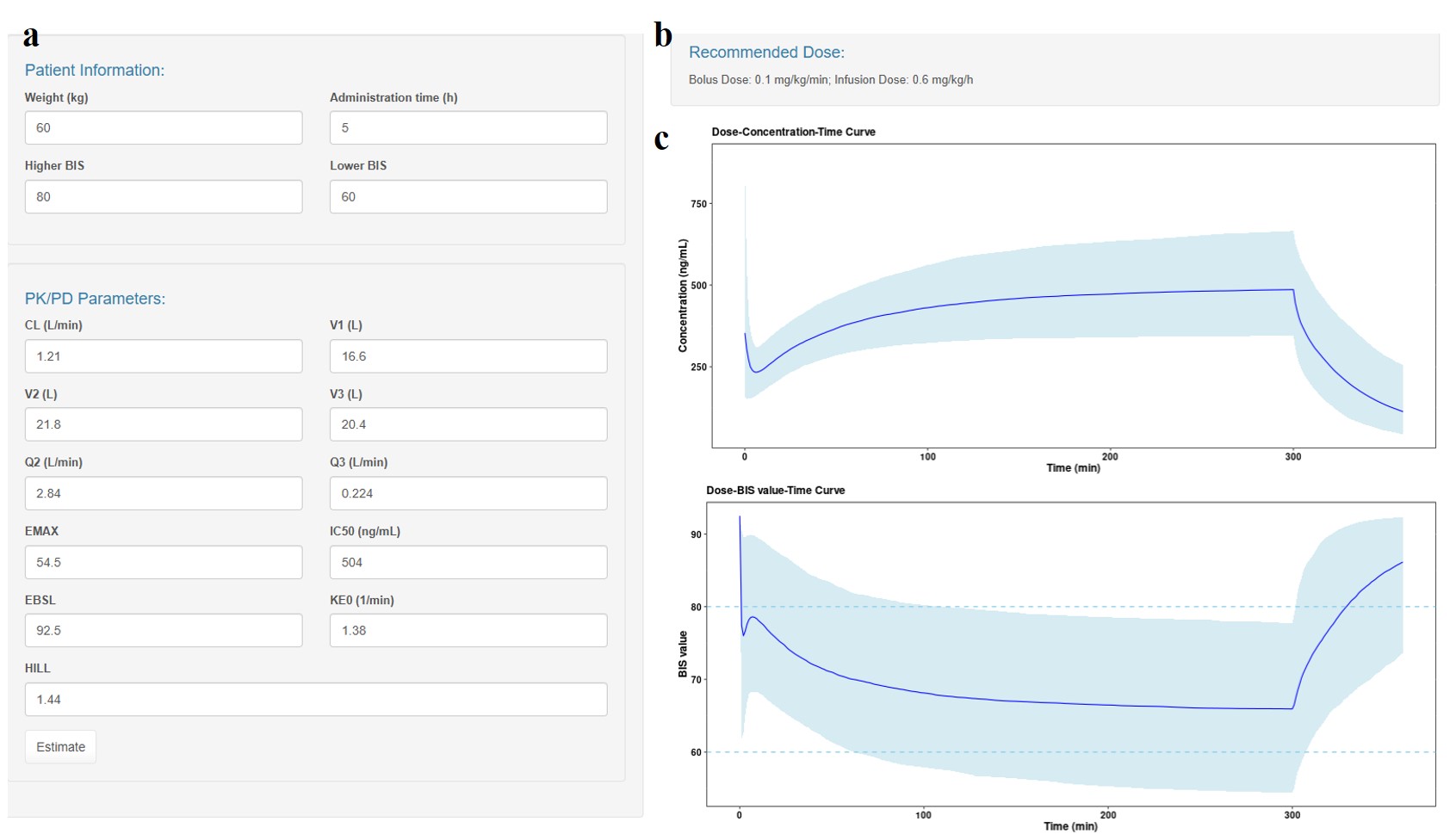

Supplement: Supplementary file 1 [file pharmaceutics-16-01122-s001.zip › pharmaceutics-3066874-supplementary materials.jpg]
